# Effect of a Synbiotic Mix on Intestinal Structural Changes, and *Salmonella* Typhimurium and *Clostridium Perfringens* Colonization in Broiler Chickens

**DOI:** 10.3390/ani9100777

**Published:** 2019-10-10

**Authors:** Zuamí Villagrán-de la Mora, Karla Nuño, Olga Vázquez-Paulino, Hugo Avalos, Javier Castro-Rosas, Carlos Gómez-Aldapa, Carlos Angulo, Felipe Ascencio, Angélica Villarruel-López

**Affiliations:** 1 Centro de Investigaciones Biológicas del Noroeste (CIBNOR), Av. Instituto Politécnico Nacional 195, Playa Palo de Santa Rita Sur 23096 La Paz, BCS, Mexico; bvillagran@pg.cibnor.mx (Z.V.-d.l.M.); eangulo@cibnor.mx (C.A.); 2 Departamento de Ciencias de la Salud, Centro Universitario de Los Altos, Universidad de Guadalajara, Av. Rafael Casillas Aceves 1200, Tepatitlán de Morelos 47620, Jalisco, Mexico; 3 Departamento de Ciencias Biomédicas, Centro Universitario de Tonalá, Universidad de Guadalajara, Nuevo Perif. Ote. 555, Ejido San José, Tateposco 45425 Tonalá, Jalisco, Mexico; karlajanette.nuno@cutonala.udg.mx; 4 Departamento de Farmacobiología, Centro Universitario de Ciencias Exactas e Ingenierías, Universidad de Guadalajara, Blvd. Gral. Marcelino García Barragán 1421, Olímpica 44430, Guadalajara, Jalisco, Mexico; olga.vazquez@academicos.udg.mx (O.V.-P.); avalossanchez@gmail.com (H.A.); 5 Área Académica de Química, Instituto de Ciencias Básicas e Ingeniería, Ciudad del Conocimiento, Universidad Autónoma del Estado de Hidalgo, Abasolo 600, Centro 42000, Pachuca de Soto, Hidalgo, Mexico; jcastro@uaeh.edu.mx (J.C.-R.); cgomeza@uaeh.edu.mx (C.G.-A.)

**Keywords:** concurrent colonization, probiotic, prebiotic, broiler, *Salmonella* Typhimurium, *Clostridium perfringens*, intestinal mucosa

## Abstract

**Simple Summary:**

In Mexico, the poultry industry uses antibiotics to improve meat production through increased feed conversion, growth rate promotion, and disease prevention. Nevertheless, due to the negative effects of antibiotic overuse and abuse, alternative strategies are required. Probiotics, Prebiotics, and Synbiotics are used as feed additives to maintain health and performance status in poultry production and have become a common method in preventing various gut diseases, but the mechanisms of how these mixtures promote animal health are still unclear. This work studies whether a Synbiotic, besides modulating the gut microbiota, can modify the intestinal mucosa ultrastructure, and if this modification can promote health conditions without affecting zootechnical parameters in broilers infected with *Salmonella* Typhimurium and *Clostridium perfringens*. Our results show that broilers treated with the Synbiotic, whether infected with pathogens or not, had healthier intestinal mucosa. The Synbiotic mix promotes structural changes in the intestinal mucosa, which in turn promotes the capacity to resist intestinal infections caused by *S*. Typhimurium and *C. perfringens* in broilers.

**Abstract:**

Synbiotics can prevent gastrointestinal infections in broilers. This work studies the effect of a Synbiotic on broilers. One-day-old male broilers were divided into groups: Control; Synbiotic; Synbiotic + *S*. Typhimurium; Synbiotic + *C. perfringens*; Synbiotic + *S*. Typhimurium + *C. perfringens*; *S*. Typhimurium; *C. perfringens*; and *S*. Typhimurium + *C. perfringens*. Histopathological analysis revealed that the Synbiotic promoted longer villi, less deep crypts, and better villi-crypt ratio. Broilers treated with the Synbiotic, infected with pathogens or not, had healthier mucosa. In groups infected with pathogens, the frequency and intensity of histopathologic lesions were lessened often in groups treated with the Synbiotic. The Synbiotic group had higher lactic acid bacteria counts than the Control group on day 39, and the isolation frequency of *S*. Typhimurium was lower (*p* < 0.05) in the Synbiotic-treated groups. On day 18, mucosa, villi, villi-crypt ratio, crypt, and feed intake were influenced by *Enterobacteriaceae*. However, on day 39 (end of the trial), those parameters were influenced by lactic acid bacteria. The Synbiotic influenced morphological modifications in the duodenal mucosa, which in turn gave the broilers the ability to resist infections caused by *S*. Typhimurium and *C. perfringens*, by inhibiting their growth and decreasing the intensity and frequency of histopathological injuries.

## 1. Introduction

*Salmonella enterica* subsp. *enterica* serovar Typhimurium (*S*. Typhimurium) and *Clostridium perfringens* (*C. perfringens*) are bacteria frequently associated with poultry. *S*. Typhimurium is a gastrointestinal pathogen and a frequent cause of food poisoning worldwide, being one of the most commonly isolated serotypes in chicken-related foodborne outbreaks [1]. It can cause morbidity and mortality in humans and poultry [2], but it can also live in poultry as a transient member of the intestinal microbial population without causing disease. Sometimes its colonisation does not affect poultry body-weight gain or performance, and this asymptomatic infection can increase the likelihood of zoonotic transmission to humans through the food chain [3]. Likewise, *C. perfringens* is a member of the normal microbiota in healthy birds but can cause myonecrotic and gastrointestinal diseases in humans and livestock, as well as in birds, under certain conditions [4]. For example, the presence of *C. perfringens* in the intestinal tract of chickens raised for meat production (broilers), even at high numbers, is not sufficient to produce necrotic enteritis. However, predisposing factors like intestinal epithelium damage, infectious bursal disease virus, high dietary levels of poorly digestible proteins, indigestible polysaccharides, feeding regime alterations, microbiota disturbances, overcrowding, and a variety of management and climatic conditions are all favorable conditions in which to develop the disease [5,6,7]. Clinical necrotic enteritis is characterized by a sudden increase in flock mortality, often without premonitory signs. Its symptoms include diarrhea, depression, reluctance to move, ruffled feathers, somnolence, decreased appetite or anorexia, huddling, and, in some cases, dribbling from the beak, dehydration, detrimental growth rate, and feeding efficiency. Notably necrotic intestinal lesions occur in the jejunum and ileum, but also in the duodenum and ceca [8,9]. Outbreaks of necrotic enteritis are common in chickens at 2–6 weeks of age, following the wane of maternal antibodies prior to the maturity of the broiler’s own immune system [8].

Subclinical necrotic enteritis can persist in broiler flocks without clinical manifestation [9], causing chronic damage to the intestinal mucosa by developing mucosal ulcerations and peripheral hyperemia [8], which leads to a decrease in digestion, absorption, and weight gain, as well as an increased feed conversion ratio and a subsequent increase in economic costs [4].

The undesired consequences of both *S*. Typhimurium and *C. perfringens* are prevented and treated by the addition of antimicrobials to the feed. However, due to the emergence of microbes resistant to antibiotics used to treat human and animal infections, the European Union decided to phase out, and finally ban, the marketing and use of antibiotics as growth promoters in feed in 2006; and the United States of America adopted these policies in 2008 [10,11]. Since the ban on growth promoting antibiotics, a rise in the incidence of subclinical necrotic enteritis and salmonellosis has become a major problem in the poultry industry, along with the subsequent decrease in animal performance and the increase of feed conversion [3,12]. Therefore, poultry farmers are looking for alternatives to control and prevent diseases in broilers, through the addition of Probiotics, Prebiotics, and Synbiotics into feed and drinking water. 

The Food and Agriculture Organization (FAO) and the World Health Organization (WHO) defined Probiotics as ‘live microorganisms that, when administered in adequate amounts, confer a health benefit on the host’ [13]. A variety of microbial species are used as Probiotics in broiler nutrition, including *Lactobacillus*, *Streptococcus*, *Bacillus*, *Bifidobacterium*, *Enterococcus*, *Aspergillus*, *Candida*, and *Saccharomyces* [14]. Prebiotics are generally defined as ‘nondigestible food ingredients that have a beneficial effect on the host by selectively stimulating the growth and/or activity of one or a limited number of bacterial species already established in the colon, and thus improving host health’ [13]. The most popular prebiotics are mannan oligosaccharides (derived from cell walls of *Saccharomyces cerevisiae*), β-glucans (derived from yeast or fungal cell walls), and fructans, inulin, levan, and the branched groups (extracted from different plants, hydrolyzed from polysaccharides, or produced by microorganism) [15]. Synbiotics are a combination of Probiotics and Prebiotics; they exhibit a synergistic relationship that positively affects the host by facilitating the implantation and survival of probiotic microorganisms in the gastrointestinal tract [16]. The use of Synbiotics in the poultry industry is based on their ability to balance the gut environment and its microbiota [17] by providing substrates for bacterial fermentation, generating antibacterial substances, competing for nutrients, modulating immune responses, and competing with pathogens for adhesion receptors on the intestinal epithelium [18]. Furthermore, Probiotics are able to modulate the intestinal permeability, mucosal immunity, and mucus layer, reducing gut permeability to molecules or bacteria and mucus degradation [14]. Based on the above, the aim of this work was to investigate the effect of a Synbiotic formulated with agave inulin as a prebiotic and *Lactobacillus rhamnosus* and *Pediococcus acidilactici* as Probiotics on duodenal morphology, content of lactic acid bacteria, and enterobacteria, as well as the growth performance in broilers of the COBBAvian48 line, infected with *S*. Typhimurium and *C. perfringens*. 

## 2. Materials and Methods

### 2.1. Treatment Preparation

Fifty milliliters of a Synbiotic mix (provided by Kurago Biotek, Jalisco, Mexico) were administered via drinking water. Each dose (1 mL) contained 7 log CFU/g of *Lactobacillus rhamnosus* HN001 and *Pediococcus acidilactici* MA18/5M and 4.5% (0.045 g) of *Agave tequilana* fructans (Patent WO2017105186 A1). 

Two pathogens were used: *S*. Typhimurium and *C. perfringens*. *S*. Typhimurium was isolated at our laboratory (from meat-food samples) and analyzed at the Mexican National Laboratory for Diagnosis and Epidemiological Reference for serotype identification according to the White–Kauffman scheme [19]. *S*. Typhimurium was subcultured, prior to broiler administration, in lactose broth with yeast extract and incubated for 24 h at 37 °C. *C. perfringens* ATCC 13124 was subcultured in thioglycolate broth and incubated for 24 h at 37 °C under anaerobic environment. 

Pathogens were separated by centrifugation (thrice at 4000 *g* for 20 min) and washed in physiological saline solution (solution of NaCl 0.8% *w*/*v*). The pellets were suspended in physiological saline solution, and the number of bacteria in the suspension was calculated using a nephelometer (DensiCHEK, Model: OA009372, bioMérieux Inc, Missouri, MO, USA).

### 2.2. Bioassay for In Vivo Evaluation of the Synbiotic Mix

This research followed the guidelines of the Institutional Animal Care and Use Committee (IACUC) and was approved by the Bioethics Committee (CUCBA) of the University of Guadalajara (Permit Number: CINV.078/15). All surgery was performed under sodium pentobarbital anesthesia (PISA Agropecuaria, Mexico), and all efforts were made to minimize broilers’ suffering.

#### 2.2.1. Housing

Two hundred and fifty-eight 1-d-old male broilers (*Gallus gallus domesticus*), line COBBAvian48 (free of growth-promoting antibiotics), were obtained from a local commercial hatchery (AVI–INC, Jalisco, Mexico) and housed in an experimental poultry shed. The surfaces of the experimental poultry shed were washed and sanitized with 200 ppm chlorine solution before the broilers arrived. Broilers were raised in stainless-steel pens (24 pens) to prevent contact between groups of birds (maximum stocking density of 30 kg/m^2^), distributed in an experimental poultry shed. They were maintained in continuous light conditions during the first two weeks, and 18 h light/6 h dark cycles for the rest of the experiment [20,21]. Room temperature was maintained at 33 ± 1 °C for the first 5 days, and then gradually reduced by 1 °C each day, until reaching 24 ± 1 °C, which was maintained for the rest of the experiment. 

All experiments were performed using a randomized complete block design, and the blocking variables were the experimental unit (pen of broiler) and the sampling time. Broilers were individually weighed and randomly divided into eight treatment groups with three replicates of each one. Each replicate was assigned to a pen physically separated from the other two of the same replicates and randomly placed in different sections of the shed. The number of birds per treatment was determined according to [22], considering the number of samples (six samples for all the treatments and three pre-samples for Control and Synbiotic treatments), number of replicas per sample (three for each treatment), and the percentage of mortality per treatment [22]. The treatments were: (1) Control group (*n* = 43); (2) Synbiotic (*n* = 35); (3) Synbiotic mix + *S*. Typhimurium (*n* = 25); (4) Synbiotic mix + *C. perfringens* (*n* = 25); (5) Synbiotic mix + *S*. Typhimurium + *C. perfringens* (*n* = 25); (6) *S*. Typhimurium (*n* = 30); (7) *C. perfringens* (n=30); and (8) *S*. Typhimurium + *C. perfringens* (*n* = 45). 

#### 2.2.2. Feeding and Vaccination

All broilers were fed ad libitum with two antibiotic-free basal diets. The starter diet was administered until the broilers were 21 days old, and the grower-finisher diet until the end of the study (6 weeks). Both diets consisted of a sorghum and soybean base, following the nutritional requirements for COBB chicken Avian48s [21] (Table 1). The traditional scheme of vaccination against avian pox, Gumboro, and Newcastle diseases was administered, the broilers weren’t vaccinated against *Salmonella* or *Clostridium*.

#### 2.2.3. Oral Administration of the Synbiotic Mix and Pathogens

The Synbiotic mix was administered in drinking water the same day broilers arrived, following the manufacturer’s instructions (open and administer birds orally). The water containers with the mix were available for 2 h, based on the average water consumption of a one-day-old broiler of, which is 1.12 mL per hour [23]. For treatment groups 3 to 8, pathogens were administered on day 17 [24], where broilers were orally challenged with 5 log CFU of *S*. Typhimurium and/or 3 log CFU of *C. perfringens* per bird through their drinking water [25]. To calculate pathogen intake, we considered that, at 17 days old, each bird consumes 25 mL of water per hour, and, therefore, water containers with the pathogen were available for 2 h [23]. There were no other drinking troughs available during the administration of the treatments (Synbiotic and pathogenic microorganisms); technicians also walked around the house to encourage drinking [26].

Birds were kept under constant observation for any sign or symptom of *Salmonella* infection or subclinical necrotic enteritis (fever, huddling, diarrhea, dejection, ruffled feathers, closed eyes, loss of appetite, and thirst).

#### 2.2.4. Slaughter and Collection of Samples

Six sampling times were scheduled with three replicas from each treatment at 18, 22, 25, 32, 36, and 39 days of life. In addition, three pre-samples were taken on broilers from Control and Synbiotic groups (at 4, 8, and 15 days of life) to know the state of its duodenal morphology, the intestinal content of Lactic Acid Bacteria and *Enterobacteriaceae*, and the absence or presence of *S*. Typhimurium and *C. perfringens* prior to the inoculation of pathogenic microorganisms. These pre-samples were not considered for statistical analysis. 

Broilers were euthanized by intraperitoneal injection of 3 mL/2.5 kg of 6.3% sodium pentobarbital (PISA Agropecuaria, Mexico). Subsequently, broilers were eviscerated, and their gastrointestinal tracts were removed under aseptic conditions. Duodenum and caeca contents were transferred into sterile containers for microbiological analysis, and duodenum tissue samples of approximately 2 cm in length were taken for histological analysis.

### 2.3. Duodenal Morphology Evaluation

Duodenum tissue samples were preserved in neutral 10% formalin and processed by the conventional paraffin inclusion method. Five-microns-thick samples were stained with hematoxylin and eosin and observed with a 4× panoramic objective (optical microscope model E200 LED, Nikon, Tokyo), and the images were analyzed using the software Motic Images Plus 2.0 (Motic, Hong Kong). Mucosal thickness, villi height, and crypt depth of nine randomly selected villi per bird group were also measured. Mucosal thickness was measured from the lamina propria to the apex of the villi, while the villi height was measured from the apex of the villi to the villi crypt junction, and the crypt depth was defined as the invagination depth between adjacent villi. Once the measurements were obtained, the villi-crypt ratio (VCR) was calculated by dividing the length of the villi by the depth of the crypt [27,28]. A qualitative analysis was also carried out using the objectives 4×, 10×, and 40×.

### 2.4. Microbiological Isolation and Enumeration

Lactic Acid Bacteria was calculated by the spread plate technique from duodenal content on Difco Lactobacilli MRS Agar plates (Cat. No. 288210, BD, Franklin Lakes, NJ, USA) and incubated at 35 °C for 36 to 48 h in a microaerobic environment. At the end of the incubation period, the colony forming units (CFU) were enumerated. Three to five colonies showing typical morphology and biochemical tests (Gram stain, catalase, and oxidase activity) were analyzed on the API 50CHL identification system (Cat. No. 50410, bioMérieux, France) to determine Lactic Acid Bacteria species [29]. *Enterobacteriaceae* were also enumerated by the surface extension technique from ceca content, plated on Petrifilm *Enterobacteriaceae* Count Plates (Cat. No. 70-2006-7092-8, 3M, MN) and incubated at 35 °C for 24 h. The isolation of *S*. Typhimurium and *C. perfringens* was also evaluated qualitatively. *S*. Typhimurium isolation was from ceca contents, using buffered peptone water (Cat. No. DF1810-17-9, BD, NJ) as a pre-enrichment solution, Tetrathionate broth base (Cat. No. 210430, BD) and Rappaport-Vassiliadis R10 (Cat. No. 218581, BD) as selective enrichment, and XLT4 Agar base (Cat. No. 223420, BD) and HE Agar (Cat. No. 254009.08, BD) for isolation. From each agar plate (XLT4 and HE Agar), three to five colonies were tested on triple-sugar-iron agar (Cat. 221038, BD), lysine-iron agar (Cat. 284920, BD), citrate agar (Cat. L007504, BD), mobility-indole-ornithine agar (Cat. 221518, BD), and urea broth (Cat. 51463-500G, Merck, Germany). Isolates showing typical *Salmonella* biochemical reactions were streaked on Tryptic Soy Agar (Cat. No. 236940, BD) and tested for slide agglutination using polyvalent serum (A-Vi Difco, Franklin Lakes, NJ) [30]. *C. perfringens* was also isolated from ceca contents, added to 20 mL of NIH Thioglycollate broth (Cat. No. 225710, BD), heated at 70 °C for 20 min in a water bath, and incubated in an anaerobic jar with a GasPack (Cat. No. 260626, BD) at 35 °C for 24 h. After incubation, an aliquot was streaked onto Tryptone-Sulfite-Cycloserine agar (TSC) (Cat. 111972, Merk) and incubated at 35 °C for 36 to 48 h in an anaerobic environment. Typical colonies were subjected to biochemical tests, Gram staining, oxidase (Cat. 261181, BD) and catalase activity (Cat. 131510, Jaloma, Mexico), Triple Sugar Iron test (Cat. 221038, BD), and milk fermentation, to confirm the presence of *C. perfringens* [31]. 

### 2.5. Growth Performance

The body weight (BW) of each bird was measured weekly, and feed intake (FI) was assessed daily by averaging the intake of each pen among the broilers present in the pen. Feed conversion ratio (FCR) was calculated as the ratio of feed consumed to weight gained, as a measure of how efficiently a bird uses energy and nutrients from the feed for growth. Flocks with a lower FCR were considered better performers [32].

### 2.6. Statistical Analysis

Data analysis (except frequency of pathogens) was examined by ANOVA or MANOVA and Tukey test. Frequency of *Lactobacillus* species and pathogens was analyzed using the non-parametric chi-squared test. Principal component analysis (PCA) and hierarchical cluster analysis (HCA) were carried out to obtain correlations between all variables (mucosal thickness, villi height, crypt depth, villi-crypt ratio, body weight, feed intake, Lactic Acid Bacteria, and *Enterobacteriaceae*) and estimate the relationships between broiler groups (Control, Synbiotic, Synbiotic + *S*. Typhimurium, *S*. Typhimurium, Synbiotic + *C. perfringens, C. perfringens,* Synbiotic + *S*. Typhimurium + *C. perfringens, S*. Typhimurium + *C. perfringens)*. All data were analyzed with Statistica 10 (TIBCO Statistica, Palo Alto, CA). Measurements were performed in triplicate. Results obtained were presented as means ± standard deviations (SD), and a *p* value less than 0.05 (*p* < 0.05) was considered significant where relevant. 

## 3. Results

The effect of a Synbiotic on the duodenal morphology, gut microbiota, and growth performance in broilers infected with *S*. Typhimurium and *C. perfringens* was investigated. At the end of the bioassay, no broiler died from *S*. Typhimurium and/or *C. perfringens* infection. Nevertheless, signs related to the colonization of these pathogens were observed. The broilers of the groups *S*. Typhimurium and *S*. Typhimurium + *C. perfringens* presented fever, huddling, diarrhea, dejection, loss of appetite, and thirst. These symptoms did not show up in the Synbiotic + *S*. Typhimurium and Synbiotic *+ S*. Typhimurium + *C. perfringens* groups after day 22. Broilers of *C. perfringens* group presented huddling, diarrhea, and dejection until day 25. The only sign of Synbiotic + *C. perfringens* group was huddling, and it was present until day 22.

### 3.1. Duodenal Morphology Differs between Broilers Fed Synbiotic Mix

**Figure 1:**
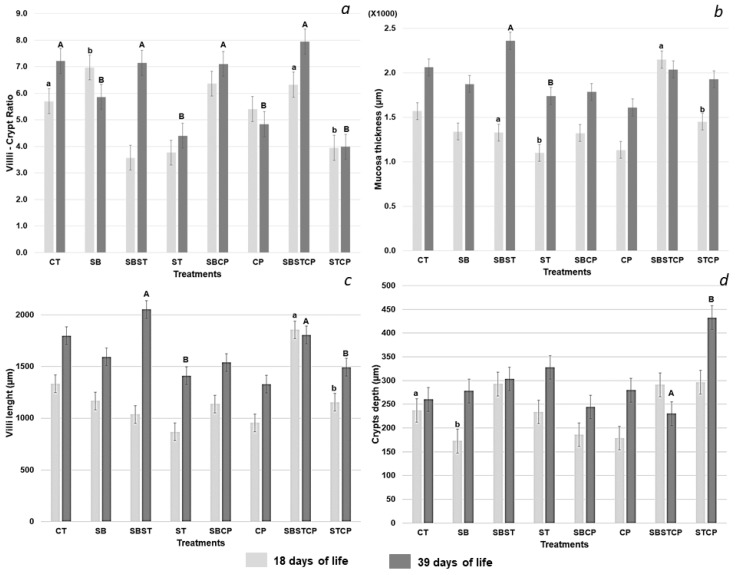
Effect of dietary supplementation with a Synbiotic mix on duodenum morphology of broilers challenged with *Salmonella* Typhimurium and *Clostridium perfringens*. (**a**) Villi–crypt ratio; (**b**) mucosa thickness (µm); (**c**) villi length (µm); and (**d**) crypts depth (µm). CT: non-challenged Control group; SB: Synbiotic; SBST: Synbiotic + *S*. Typhimurium; ST: *S*. Typhimurium; SBCP: Synbiotic + *C. perfringens;* CP: *C. perfringens;* SBSTCP: Synbiotic + *S*. Typhimurium + *C. perfringens*; STCP: *S*. Typhimurium + *C. perfringens*. Means in the same row with different superscripts (a–b or A–B) differ (MANOVA *p* < 0.05).

Duodenal morphology of the broilers, based on the VCR, villi length, crypt depth, and mucosa thickness, was evaluated on day 18 (24 h after inoculation with *C. perfringens* and *S*. Typhimurium) and on day 39. The average value of the VCR in the pre-samplings was 3.9 ± 0.9 for birds in the Control group and 5 ± 1.2 for those in the Synbiotic group. The VCR of the Synbiotic group was significantly higher (*p* < 0.05) compared to the Control group on day 18 (average of 7 ± 0.47 vs. 5.7 ± 0.47); however, at the end of the experiment (day 39), the VCR of the Control group was higher than the Synbiotic group’s (average of 7.2 ± 0.47 vs. 5.9 ± 0.47). On day 39, in groups inoculated with the pathogens, the VCR was higher (*p* < 0.05) in groups treated with the Synbiotic mix when compared to the *S*. Typhimurium group (average of 7.1 ± 0.47 vs. 4.4 ± 0.47), the *C. perfringens* group (average of 7.1 ± 0.47 vs. 4.8 ± 0.47), and the *S*. Typhimurium + *C. perfringens* group (average of 7.9 ± 0.5 vs. 4 ± 0.47) (Figure 1a) (File S2).

Mucosal thickness of the broilers in the Synbiotic + *S*. Typhimurium group was significantly thicker (*p* < 0.05) when compared to the *S*. Typhimurium group on day 18 (average of 1328 ± 95.21 μm vs. 1100 ± 95.21 μm) and on day 39 (average of 2359 ± 95.21 μm vs. 1738 ± 95.21 μm) and to the *S*. Typhimurium + *C. perfringens* group at day 18 (average of 2148 ± 95.21 μm vs. 1450 ± 95.21 μm) (Figure 1b). The average value of mucosa thickness in pre-samples was 735 ± 191.3 μm in the Control group and 623 ± 61.3 μm in the Synbiotic group (Figure 1b) (File S2).

Villi length average in pre-samples was 594 ± 91.7 μm in the Control group and 528 ± 50.8 μm in the Synbiotic group. Villi length following Synbiotic treatment was significantly longer (*p* < 0.05) in groups inoculated with the pathogens compared to respective Controls on day 39 of treatment in both the *S*. Typhimurium (average of 2055 ± 85.79 μm vs. 1410 ± 85.79 μm), and *S*. Typhimurium + *C. perfringens* (average of 1807 ± 85.79 μm vs. 1494 ± 85.79 μm) groups (Figure 1c) (File S2). 

The average of crypt depth in pre-samples was 140 ± 20.2 μm for broilers in the Control group and 95 ± 17.8 μm in the Synbiotic group. Crypt depth following Synbiotic treatment was smaller compared to respective Control, in the *S*. Typhimurium + *C. perfringens* group on day 39 (230 ± 24.98 μm vs. 433 ± 24.98 μm) (Figure 1d) (File S2). 

There were no significant differences observed in any of the morphological features mentioned above between the *C. perfringens* and Synbiotic + *C. perfringens* groups (Figure 1a–d) (File S2). 

**Figure 2:**
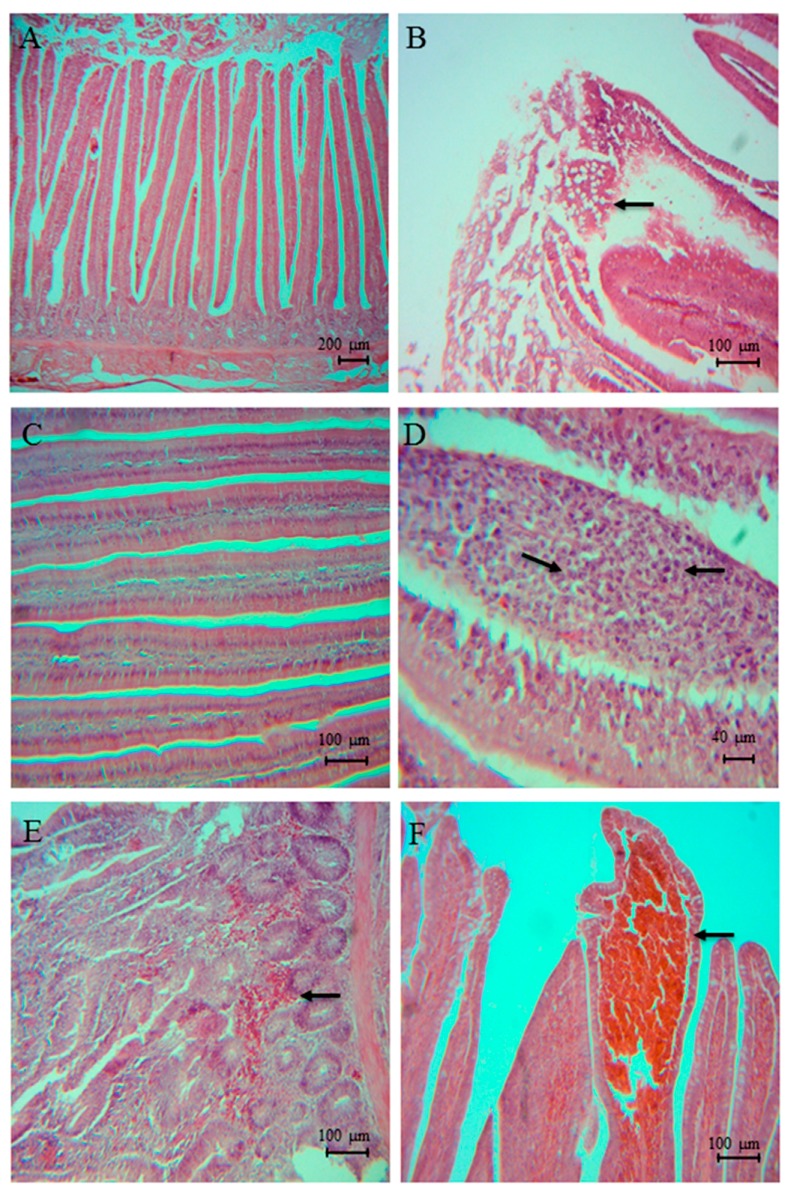
Histopathological lesions in duodenum of broilers challenged with *Salmonella* Typhimurium and *Clostridium perfringens*. (**A**) Epithelium without apparent lesions (4×), (**B**) calciform cells hyperplasia (10×), (**C**) villi without apparent lesions (10×), (**D**) lymphocyte infiltration (40×), (**E**) hemorrhagic villi (10×), and (**F**) congested villi (10×).

No histopathological lesions were found in the non-pathogen Control or Synbiotic groups (Figure 2A,C). Broilers treated with *S*. Typhimurium showed multifocal epithelium hyperplasia with mucosa degeneration (Figure 2B), lymphocyte infiltration, and congested villi from day 22 until the end of the experiment, but those broilers receiving concurrent Synbiotic treatment showed no sign of lesions on day 32. Similarly, broilers treated with *C. perfringens* showed lymphocyte infiltration (Figure 2D), hemorrhagic villi (Figure 2E), and discreet multifocal necrosis of the mucosa from day 25 until the end of the experiment, whereas broilers receiving concurrent Synbiotic treatment showed no sign of lesions from day 32 and no sign of mucosal necrosis. Broilers receiving *S*. Typhimurium + *C. perfringens* exhibited all the above described lesions, as well as congested villi (Figure 2F), a feature not observed with *S*. Typhimurium or *C. perfringens* treatment alone, from day 22 until the end of the experiment. Broilers receiving concurrent Synbiotic treatment only exhibited these lesions on day 22 and 25, with no hemorrhagic villi observed.

### 3.2. Microbiological Isolation and Enumeration from Intestinal Contents of Broilers Challenged with S. Typhimurium and C. Perfringens

Lactic Acid Bacteria were isolated from the duodenum, and the average load in the pre-samples (4, 8, and 15 days of life) was 5.3 ± 1.1 log CFU/g for the Control group and 5 ± 1.4 log CFU/g for the Synbiotic group. No significant differences (*p* > 0.05) in Lactic Acid Bacteria counts were observed among treatments until day 36, when broilers infected with *S*. Typhimurium had a significantly lower Lactic Acid Bacteria count (*p* < 0.05) (4.6 ± 1 log CFU/g) than in other groups (Synbiotic <6.5 ± 0.7 log CFU/g>, Synbiotic + *C. perfringens* <6.3 ± 0.1 log CFU/g> and Synbiotic + *S*. Typhimurium + *C. perfringens* <6.5 ± 0.1 log CFU/g>), and on day 39, significantly higher counts (*p* < 0.05) were observed in the Synbiotic group (6.5 ± 0.4 log CFU/g) compared to the Control group (4.1 ± 1.6 log CFU/g), the Synbiotic + *S*. Typhimurium group (3.9 ± 0.3 log CFU/g), and the Synbiotic + *C. perfringens* group (3.9 ± 0.3 log CFU/g) (Table 2) (File S1).

A total of 110 strains of Lactic Acid Bacteria were identified from broiler groups both with and without Synbiotic treatment. Eight *Lactobacillus* species (*L. delbrueckii, L. fermentum, L. acidophilus, L. brevis, L. crispatus, L. plantarum, L. lactis*, and *L. salivarius*) were found in all treatment groups. In treatments with the Synbiotic mix, *L. rhamnosus* and *L. curvatus* were present. In broilers without the Synbiotic mix, only *L. mesenteroides, L. pentosus*, and *L. buchneri* were found.

*Enterobacteriaceae* were isolated from the ceca content of the broilers. *Enterobacteriaceae* counts across the Synbiotic group were lower than that of the Control group, but this did not reach statistical significance until day 32, where the Control (8.1 ± 0.3 log CFU/g) and *S*. Typhimurium (8.1 ± 0.1 log CFU/g) groups exhibited higher (*p* < 0.05) counts compared to the other groups (6–7.9 ± 0.4 log CFU/g). The *C. perfringens* group presented the lower counts (6 ± 0.3 log CFU/g), with similar trends observed until the end of the experiment (File S1).

**Figure 3:**
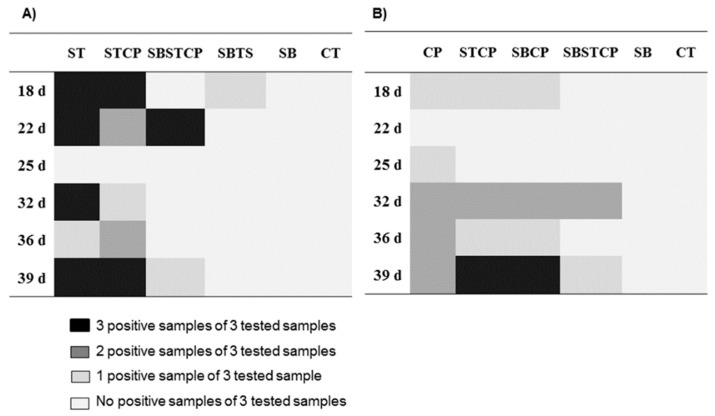
Effect of dietary supplementation with a Synbiotic mix on the isolation of the inoculated *Salmonella* Typhimurium and *Clostridium perfringens* from ceca contents of broilers. (**A**) Isolation of the inoculated *Salmonella* Typhimurium and (**B**) isolation of the inoculated *Clostridium perfringens*. CT: non-challenged Control group; SB: Synbiotic; SBST: Synbiotic + *S*. Typhimurium; ST: *S*. Typhimurium; SBCP: Synbiotic + *C. perfringens*; CP: *C. perfringens*; SBSTCP: Synbiotic + *S*. Typhimurium + *C. perfringens*; and STCP: *S*. Typhimurium + *C. perfringens*.

The isolation of the inoculated *S*. Typhimurium and *C. perfringens* was investigated in the cecal contents in groups inoculated with the pathogens, treated or non-treated with the Synbiotic mix. There were no *S*. Typhimurium or *C. perfringens* isolates in the samples taken before the administration of the pathogens. The isolation of the inoculated *S*. Typhimurium was significantly lower (*p* < 0.05) in the Synbiotic treatment groups (Synbiotic + *S*. Typhimurium and Synbiotic + *S*. Typhimurium + *C. perfringens*) compared to the *S*. Typhimurium, *C. perfringens*, and *S*. Typhimurium + *C. perfringens* groups. There was no difference in the isolation of the inoculated *C. perfringens* between groups treated or untreated with the Synbiotic mix (*p* > 0.05) (Figure 3) (File S1).

### 3.3. Effect of the Synbiotic on Growth Performance

Body weight (BW), feed intake (FI), and feed conversion ratio (FCR) were also evaluated every week during the study (6 weeks). There was no significant difference in body weight with or without Synbiotic supplementation in birds (*p* > 0.05) (Table 3). Likewise, feed intake and FCR did not differ between treatment groups (File S3). 

### 3.4. Principal Component Analysis

**Figure 4:**
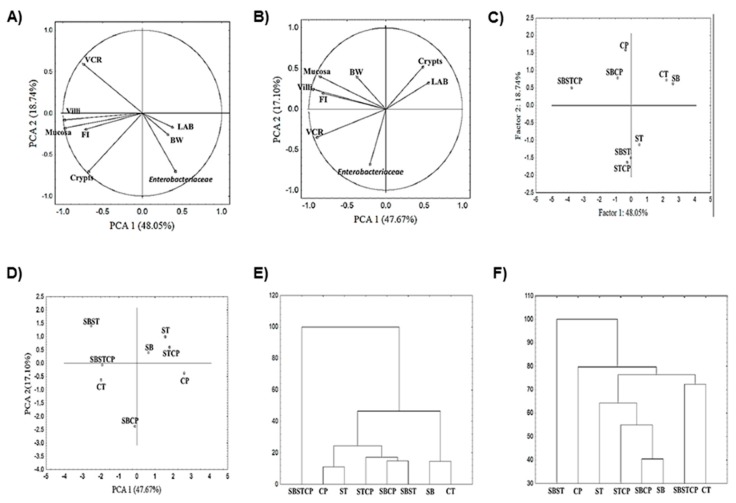
Principal component analysis (PCA) plots of broilers treated with a Synbiotic mix. (**A**) Location of different variables at 18 days; (**B**) location of different variables at 39 days; (**C**) location of different treatments at 18 days; (**D**) location of different treatments at 39 days; (**E**) dendrogram of hierarchical cluster analysis at 18 days; (**F**) dendrogram of hierarchical cluster analysis at 39 days. BW: body weight, VCR: villi-crypt ratio, FI: feed intake, LAB: Lactic Acid Bacteria. CT: non-challenged Control group; SB: Synbiotic mix; SBST: Synbiotic mix + *S*. Typhimurium; ST: *S*. Typhimurium; SBCP: Synbiotic mix + *C. Perfringens*; CP: *C. Perfringens*; SBSTCP: Synbiotic mix + *S*. Typhimurium + *C. Perfringens*; and STCP: *S*. Typhimurium + *C. Perfringens*.

Principal component analysis (PCA) was applied to determine any pattern recognition between variables and treatments in addition to a comparison between the behavior of variables in broilers after 24 h of pathogenic administration (day 18) and at the end of trial (day 39). Principal components (PC) 1 and 2 had a high percentage of the total variance at 48% and 19%, respectively, on day 18, and 48% and 17%, respectively, on day 39. Parameters are presented in Figure 4A,B and the graph location of working groups are shown in Figure 4C,D. Thus, in a scatter plot of the parameter score values projected in the PC1 and PC2 planes (day 18), mucosa (−0.97), villi (−0.98), VCR (−0.74), crypt (−0.67) and feed intake (−0.71) were influenced by the presence of *Enterobacteriaceae* (0.41), proceeding from negative to positive values of PC1 (Figure 4A). On day 39, mucosa (−0.85), villi (−0.94), VCR (−0.89) and feed intake (−0.71) were influenced by the presence of Lactic Acid Bacteria (0.55), proceeding from negative to positive values of PC1 (Figure 4B). As indicated in Figure 4C,D, PCA can distinguish between broiler groups with or without Synbiotic treatment at both time-points. PC1 and PC2 showed a separation between broiler groups. According to the coordinate’s factor, on day 18, Control (2.20) and Synbiotic mix (2.60) have a closer relationship. Synbiotic mix + *S*. Typhimurium + *C. perfringens* (−3.75) showed a higher difference from both Control and Synbiotic mix compared to Synbiotic mix + *S*. Typhimurium (−0.04), Synbiotic mix + *C. perfringens* (−0.86), *S*. Typhimurium (0.49), *C. perfringens* (−0.37) and *S*. Typhimurium + *C. perfringens* (−0.26). On day 39, Control (−1.99) and Synbiotic mix + *S*. Typhimurium + *C. perfringens* (−1.91) exhibited a close relationship and were different with respect to *S*. Typhimurium (1.54), *S*. Typhimurium + *C. perfringens* (1.77) and *C. perfringens* (2.59), proceeding from negative to positive values of PC1 in both dates.

Additionally, hierarchical cluster analysis (HCA) was carried out and is displayed as a dendrogram in Figure 4E,F. This analysis indicated that the broilers within the same group are more like each other than samples in different groups. On day 18, Synbiotic mix + *S*. Typhimurium + *C. Perfringens* (first cluster) is clearly discernible from the other groups. A second cluster consists of *C. Perfringens*, *S*. Typhimurium, *S*. Typhimurium + *C. Perfringens*, Synbiotic mix + *C. Perfringens*, and Synbiotic mix + *S*. Typhimurium. The third cluster includes Synbiotic mix and Control. On day 39, differences in cluster grouping were observed. The first cluster is for Synbiotic mix + *S*. Typhimurium; the second cluster includes *C. Perfringens*, *S*. Typhimurium, *S*. Typhimurium + *C. Perfringens*, Synbiotic mix + *C. Perfringens* and Synbiotic mix; and the third cluster consists of Synbiotic mix + *S*. Typhimurium + *C. Perfringens* and Control. 

## 4. Discussion

The presence of *C. perfringens* and *S*. Typhimurium in broilers can cause significant economic losses. These bacteria have traditionally been controlled using antibiotics, but due to the negative effects of antibiotic overuse and abuse, alternative strategies are required. Probiotics, Prebiotics, and Synbiotics are used as feed additives to maintain health and performance status in poultry production and have become a common method in preventing various gut diseases [33]. *L. rhamnosus* is one of the most widely used Probiotic strains, has the potential to be a good Probiotic choice thanks to its strong adhesive capacity, and is also able to enhance immunity [34,35,36]. It was shown to protect animals against gastrointestinal pathogens like *E. coli* O157:H7 [37] and *S*. Typhimurium [38]. *P. acidilactici* exert antagonism against other microorganisms like *Clostridium* spp. and *Salmonella,* primarily through the production of lactic acid, in addition to the production of antimicrobial peptides known as pediocins [39,40,41]. Fructans enhances the population of beneficial bacteria, such as bifidobacteria and lactobacilli, and suppresses levels of pathogenic bacteria, such as *C. perfringens* and *E. coli*, in the intestine of broilers [15]. *Agave tequilana Fructans* promotes the growth of probiotic bacteria such as *Lactobacillus salivarius* and *Enterococcus faecium*, and its Prebiotic effect surpasses chicory inulin [42].

In the current study, the intestinal tract mucosa of broilers infected with *S*. Typhimurium and *C. perfringens* and treated with the Synbiotic formulation was healthier, with higher VCR than their controls (*S*. Typhimurium, *C. perfringens*, and *S*. Typhimurium + *C. perfringens* groups). Colonization with the probiotic *Lactobacillus rhamnosus* promoted these ultrastructural changes by increasing epithelial cell turnover regulation [43]. Alternatively, the combination of the prebiotic (*A. tequilana* fructans) with Lactic Acid Bacteria in the intestine, could facilitate the implantation of other native Lactic Acid Bacteria species with a protective effect [44]. Some authors have reported that Probiotics such as *L. johnsonii* BSNE [45] and Synbiotics like *E. faecium* DSM 3530 and chicory prebiotic [46], or *B. Subtilis* and xylooligosaccharide [47] can enhance intestinal development. 

It is known that shorter villi and deeper crypts are associated with the presence of bacterial toxins as the mucosa attempts to restore the epithelial cells affected by constant damage [48]. Concurrently, we found these shorter villi and deeper crypts in the groups infected with pathogens compared to the Control group, but not in pathogen groups receiving Synbiotic treatment. 

The histological examination of the duodenum revealed lesions relating to subclinical necrotic enteritis and *Salmonella* colonization (epithelium hyperplasia, mucosa degeneration, lymphocyte infiltration, congested, and hemorrhage villi) in groups inoculated with the pathogens, but the frequency and intensity of lesions were lower in the Synbiotic-treated groups when compare to respective Controls. Our results indicate that the ultrastructural changes promoted by the Synbiotic mix could increase the resistance capacity of broilers to intestinal infections caused by *S*. Typhimurium and *C. perfringens*, as reported by Stanley et al. (2012) [49].

Host responses to infectious agents are often regulated through phosphorylation [50], and Prebiotics may impact host signaling affecting mucosal inflammation, independently of the presence of microbes [51]. Prebiotics were also shown to directly mediate changes in barrier function [52]. Synbiotics prevent infections by stimulating the microbiota of the host [53]. 

Lactic Acid Bacteria can alter gut dynamics and physiologic processes related to intestinal functions [18]. They improve bird nutrition by helping with the digestion process and synthesizing nutrients, which also stimulates the intestinal epithelium and reduces intestinal diseases by preventing the colonization of pathogenic microorganisms.

In our study, birds treated with the Synbiotic mix tended to show higher Lactic Acid Bacteria counts than those not treated (challenged or not with pathogens). However, these differences were not significant until 39 days of age, when birds of the Synbiotic group showed a higher Lactic Acid Bacteria count (6.5 log_10_ CFU/g) than the Control group (4.1 log_10_ CFU/g), which is comparable to a previous study, where counts of 6.68 log_10_ CFU/g were recorded in the ileum content of broilers treated with a commercial Synbiotic mix [54]. 

The fact that no differences were found prior to day 39 can be related to the development and natural establishment of the intestinal microbiota of broilers. It was reported that the microbial community of the small intestine is abundant in fecal streptococci and coliforms for the first 40 days of life and then lactobacilli become established and dominant [18,55]. Furthermore, the timing of probiotic administration may influence the beneficial effect. Nakphaichit et al. [56] administered *Lactobacillus reuteri* during the first week post-hatch, and, as in this study, they found no significant effects on day 18. However, on day 42, delayed effects were shown through the increase in diversity and abundance of *Lactobacillus*. Finding higher Lactic Acid Bacteria counts before 40 days of life in the Synbiotic group suggested that Synbiotic treatment introduced the conditions for enrichment of *Lactobacillus* species and modification of intestinal microbiota of broilers.

A broiler’s intestinal microbiota is composed of three major genera, *Lactobacillus*, *Clostridium*, and *Bacteroides*. A balanced intestinal microbiota and high microbial diversity (especially in lactobacilli) enhance resistant to infection [32,56,57]. *Lactobacillus* are sensitive to stress and tend to decrease when a bird is under stress, disturbing the microbiota balance and facilitating the development of gastrointestinal infections. Synbiotics selectively stimulate the growth and/or activity of those *Lactobacillus* [58,59]. 

In our study, *L. rhamnosus* and *L. curvatus* were only found in broilers treated with the Synbiotic mix, while *L. pentosus, L. buchneri*, and *L. mesenteroides* were found exclusively in broilers that did not receive the Synbiotics. *L. rhamnosus* improves growth performance, meat quality, ammonia emission, and intestinal microbiota in chickens [60]. *Lactobacillus curvatus* display antimicrobial activity against *Klebsiella, E. coli*, and *Staphylococcus aureus* [61]. *Lactobacillus plantarum*, which is associated with weight loss, and *L. acidophilus* and *L. fermentum*, which are associated with weight gain, were isolated from both broiler groups [62]. 

Probiotics and Prebiotics can influence the intestinal microbiota [15,63]; therefore, to evaluate the effect of the Synbiotic mix on the Gram-negative gut microbiota of broilers, Enterobacteriaceae counts were determined. However, these remained relatively constant throughout the six-week trial period, which is similar to a previous study where differences were observed only after 35 days [59]. 

The control of *Salmonella* is one of the major tasks in poultry production to ensure food safety, and the modulation of intestinal microbiota with Prebiotics, Probiotics, and Synbiotics in broilers has reduced farms contaminated with *Salmonella* [64]. In this study, the isolation of the inoculated *S*. Typhimurium was significantly lower in the group receiving *S*. Typhimurium and the Synbiotic mix, compare to *S*. Typhimurium alone. Previous research has shown a reduction of 58% of *Salmonella* in birds fed with *B. subtilis* [3], as well as a decreased in *Salmonella* numbers in the large and small intestine and in feces [65]. 

*C. perfringens* represents a hazardous risk to a broiler’s health when it is present in its small intestine. Its growth in the gastrointestinal tract depends on favorable conditions and subsequently extends pathogenicity [66]. During this study, *C. perfringens* was recovered from the ceca contents of inoculated broilers 24 hours post-inoculation and throughout the trial (39 days), but it was not recovered in non-inoculated groups. Not all broilers from which the pathogen was recovered showed signs of subclinical necrotic enteritis. However, it was reported that there is no direct relationship between the number of *C. perfringens* positive cells present in the gastrointestinal tract and the development of the disease [9,67]. 

The alteration of the microbiota, especially the decrease in the number of Lactic Acid Bacteria, could help in the development of the disease [68]. Qing et al. proved that *Lactobacillus johnsonii* BS15 can prevent subclinical necrotic enteritis caused by *C. perfringens* through adjusting the intestinal microbiota composition [45]. Wang et al. concluded that feed supplementation with *L. johnsonii* BS15 may prevent subclinical necrotic enteritis by the enhancement of small intestinal immunity [69].

It was demonstrated that the use of additives in the diet of broilers affects the intestinal microbial balance and subsequently improves the growth performance and reduces the mortality rate [70]. In the present study, no differences in the body weight of the broilers were found (*p* > 0.05). Weight gain discrepancies may be due to the use of diverse strains of Probiotics, different prebiotic fibers, environmental conditions, and the genetic lines of the chickens. Furthermore, Synbiotic additives can be more effective under stress conditions, extreme temperatures, crowding, and poor management conditions [71], which were avoided during this study.

Multivariate tools such as Principal Components Analysis and Hierarchical Cluster are used in poultry research. These uses include analyzing performance and measuring carcass traits [72,73]; measuring morphostructural traits [74]; observing how the laying cycle can be divided and what the relationships of the breeding values of egg production are between the partial periods and the total period [75]; and comparing rep-PCR genomic fingerprinting methods for differentiation of fecal *Escherichia coli* from humans, poultry, and wild birds [76]. However, to our knowledge, this is the first time that it has been used to evaluate the effect of a Synbiotic mix on broilers’ health and growth. 

Our analysis revealed different patterns of variables and treatments on day 18 and 39. On day 18, mucosa thickness, villi, crypt depth, villi-crypt ratio, and feed intake were influenced by the presence of *Enterobacteria*, whereas at the end of the trial, the same parameters were influenced by the Lactic Acid Bacteria. According to Cisek and Binek [77], the intestinal microbiota affects, either negatively or positively, intestinal maturation and development.

The hierarchical cluster analysis grouped the broilers from the different treatments into three clusters on day 18 and three on day 39. On day 18, the broilers of the cluster Synbiotic mix + *S*. Typhimurium + *C. Perfringens* were characterized by showing the thickest intestinal mucosa, followed by the cluster formed by Synbiotic mix and Control, and finally the cluster formed by *C. Perfringens*, *S*. Typhimurium, *S*. Typhimurium + *C. Perfringens*, Synbiotic mix + *C. Perfringens*, Synbiotic mix + *S*. Typhimurium groups, whose mucosa was the thinnest compared to that of the two previous clusters. On day 39, the grouping of the clusters was different, being the one formed by Synbiotic mix + *S*. Typhimurium the one with the thickest mucosa, followed by the cluster Synbiotic mix + *S*. Typhimurium + *C. Perfringens* and Control, and the cluster with the thinnest mucosa was formed by *C. Perfringens*, *S*. Typhimurium, *S*. Typhimurium + *C. Perfringens*, and Synbiotic mix + *C. Perfringens* and Synbiotic mix. Probiotics are regarded as modifying agents of the intestinal-wall thickness due to the resulting elimination of harmful bacteria [59].

## 5. Conclusions

The Synbiotic composed of *Lactobacillus rhamnosus* HN001, *Pediococcus acidilactici* MA18/5M, and *Agave tequilana fructans* influenced morphological modifications (longer villi and less-deep crypts) in the duodenal mucosa, which in turn gave the broilers the ability to resist infections caused by *S*. Typhimurium and *C. perfringens*, by inhibiting the growth of *S*. Typhimurium and decreasing the intensity and frequency of histopathological injuries associated with subclinical necrosis caused by *C. perfringens*. However, the productive parameters (body weight, feed intake, and feed conversion ratio) were not modified with the administration of the Synbiotic.

On the other hand, the broilers treated with the Synbiotic showed a tendency toward having higher counts of Lactic Acid Bacteria throughout the bioassay, and different strains of groups with and without Synbiotic treatment were identified.

## Figures and Tables

**Table 1:** Ingredients and bromatological composition of the basal diets administered to broilers.

	Start	Grower-Finisher
**Ingredients(g/Kg)**		
Sorghum	640	690
Soybean	255	210
Vitamin and mineral Premix	80	70
Sunflower oil	25	30
**Bromatological composition (%)**		
Dry material	94.91	90.5
Humidity	5.09	9.5
Ashes	7.10	6.3
Crude protein	21.91	18.6
Ether extract	6.02	6.13
Crude fiber	2.90	3.1
Nitrogen free elements	56.98	56.37
Calcium	0.98	0.74
Phosphorus	0.48	0.40
Metabolizable Energy (Mcal/kg)	3.08	3.23

**Table 2:** Effect of dietary supplementation with a Synbiotic mix on duodenal Lactic Acid Bacteria counts of broilers challenged with *Salmonella* Typhimurium and *Clostridium perfringens*.

	Mean Lactic Acid Bacteria (Log_10_ CFU/g) ± Standard Deviation					
Treatment ^1^/Days	18 d	22 d	25 d	32 d	36 d	39 d
CT	5.8 ± 0.7	6.3 ± 0.4	5.8 ± 1.7	5.3 ± 0.9	5.6 ± 0.4 ^ab^	4.1 ± 1.6 ^a^
SB	5.0 ± 1.9	6.2 ± 1.0	5.6 ± 1.3	5.2 ± 1.3	6.5 ± 0.7 ^a^	6.5 ± 0.4 ^b^
SBST	6.7 ± 0.3	5.6 ± 0.4	5.1 ± 1.2	4.4 ± 1.0	5.5 ± 0.2 ^ab^	3.9 ± 0.3 ^a^
ST	5.0 ± 1	5.3 ± 1	4.0 ± 0.6	4.5 ± 1.2	4.6 ± 1.0 ^b^	5.4 ± 0.8 ^ab^
SBCP	4.7 ± 1.6	4.9 ± 1.6	3.7 ± 0.8	5.9 ± 0.2	6.3 ± 0.1 ^a^	3.9 ± 0.3 ^a^
CP	5.9	5.3	4.2 ± 1.9	4.5 ± 1.2	5.2 ± 0.3 ^ab^	5.4 ± 0.3 ^ab^
SBSTCP	4.4 ± 0.7	6.0 ± 0.7	4.9 ± 0.4	5.0 ± 0.8	6.5 ± 0.1 ^a^	5.2 ± 0.6 ^ab^
STCP	4.8 ± 1.6	5.7 ± 1.6	4.8 ± 0.8	4.2 ± 0.2	5.2 ± 0.1 ^ab^	4.9 ± 0.3 ^a^

^1^ CT: Control group; SB: Synbiotic mix; SBST: Synbiotic mix + *S*. Typhimurium; ST: *S*. Typhimurium; SBCP: Synbiotic mix + *C. perfringens;* CP: *C. perfringens*; SBSTCP: Synbiotic mix + *S*. Typhimurium + *C. perfringens;* STCP: *S*. Typhimurium + *C. perfringens*. Mean values of three replicates with different letter (a,b) in the same column are significantly different (*p* < 0.05).

**Table 3:** Effect of dietary supplementation with a Synbiotic mix on the body weight of birds challenged with *Salmonella* Typhimurium and *Clostridium perfringens*.

Treatment ^1^ Days	Mean Body Weight (g) ± Standard Deviation						
	1 d	7 d	14 d	21 d	28 d	35 d	42 d
CT	43 ± 2.9	147 ± 11.8	424 ± 35.8	738 ± 74.2 ^a^	1148 ± 125.2	1734 ± 199.2	2416 ± 330
SB	44 ± 2.9	162 ± 13.2	432 ± 31.4	799 ± 54.5	1175 ± 70	1848 ± 93.7	2550 ± 26.4
SBST	44 ± 4.3	162 ± 2	448 ± 50.3	779 ± 96.6	1185 ± 121.8	1740 ± 197	2588 ± 106.8
ST	44 ± 3.1	152 ± 13.9	437 ± 63.2	747 ± 152.6	1071 ± 236.4	1836 ± 185.7	2491 ± 168.2
SBCP	44 ± 3.3	164 ± 18.5	460 ± 52.8	847 ± 115.8 ^b^	1306 ± 83.3	1969 ± 175.6	2693 ± 135
CP	44 ± 3.1	161 ± 12.4	465 ± 40.4	800 ± 70.3	1271 ± 114.2	1936 ± 218.7	2696 ± 360
SBSTCP	44 ± 2.7	153 ± 12.2	440 ± 39.6	812 ± 71.5	1206 ± 142.7	1860 ± 204.3	2295 ± 210.7
STCP	46 ± 4.6	155 ± 16.9	438 ± 51.2	794 ± 96.4	1211 ± 166.5	1780 ± 147	2453 ± 182.6

^1^ CT: Control group; SB: Synbiotic mix; SBST: Synbiotic mix + *S*. Typhimurium; ST: *S*. Typhimurium; SBCP: Synbiotic mix + *C. perfringens*; CP: *C. perfringens*; SBSTCP: Synbiotic mix + *S*. Typhimurium + *C. perfringens*; and STCP: *S*. Typhimurium + *C. perfringens*. Mean values of three replicates with different letter (a,b) in the same column are significantly different (*p* < 0.05).

## Supplementary Materials

The following are available online at https://www.mdpi.com/2076-2615/9/10/777/s1. File S1: Raw data from microbiological isolation and enumeration. File S2: Raw data from Duodenal morphology evaluation. File S3: Raw data from growth performance evaluation.
